# Intermediate-Term Survivorship of Total Hip Arthroplasty With a Proximally Coated Tapered-Wedge Femoral Stem: A Retrospective, Multi-Center Registry Review

**DOI:** 10.7759/cureus.36623

**Published:** 2023-03-24

**Authors:** David W Fawley, Sean Croker, John F Irving, Michael L Swank

**Affiliations:** 1 Clinical Research, DePuy Synthes, Warsaw, USA; 2 Orthopaedic Surgery, Connecticut Orthopaedics, Hamden, USA; 3 Orthopaedic Surgery, Cincinnati Orthopaedic Research Institute, Cincinnati, USA

**Keywords:** tapered wedge stem, cementless, registry, survivorship, total hip arthroplasty

## Abstract

Background: Short tapered-wedge stems have been used frequently over the past decade, but long-term follow-up data are not readily available in the literature.

Methods: A retrospective outcomes review was conducted to assess survivorship and clinical outcomes for the TRI-LOCK® Bone Preservation Stem (TRI-LOCK BPS; DePuy Synthes, Warsaw, IN, USA), a proximally coated, tapered-wedge femoral stem.

Results: In a cohort of 2,040 hips, Kaplan-Meier survivorship estimates (95% CI {confidence interval}; N with further follow-up, where N is the number of hips remaining at each post-operative interval), with survivorship defined as no revision of any component for any reason were 96.6% (92.8%,98.4%; 45) at eight years under the clinical assumption and 98.6% (97.9%,99.1%; 90) at 14 years under the registry assumption. With survivorship defined as stem revision for any reason, estimates were 97.7% (93.7%,99.2%; 45) at eight years under the clinical assumption and 99.2% (98.6%,99.5%; 90) under the registry assumption. Mean Harris Hip Scores and WOMAC scores were 90.08 and 21.98, respectively, at 10 years postoperatively.

Conclusion: Our evaluation demonstrates excellent construct and stem survivorship and clinical outcomes at intermediate-term postoperative follow-up.

## Introduction

Total hip arthroplasty (THA) is one of the most successful surgeries in the world [1]. Successful THA helps patients regain mobility and range of motion and reduce pain, which often greatly enhances quality of life [2-4]. A large number of THAs today are performed as cementless procedures, and appropriate implant design is required for adequate rotational stability and biological bone ingrowth and on-growth [5,6]. Additionally, within the last decade there has been increasing interest in minimally invasive techniques [7-9] and short stems [10-15] that are often optimized for use in these procedures. Many published reports have shown success for short stems regarding survivorship, particularly related to aseptic loosening [11], but there are conflicting reports regarding complications with use of short stems in minimally invasive THA. Some studies have shown short tapered stems to be helpful in reducing complications [16-18], while other studies have reported no difference [19] or increased complications [20,21].

The original TRI-LOCK® stem was introduced in 1981 as the first proximally coated tapered-wedge hip stem. Released in 2008, the TRI-LOCK® Bone Preservation Stem (TRI-LOCK BPS; DePuy Synthes, Warsaw, IN, USA) was designed to help provide consistent implant seating based on a simple reproducible surgical technique and to achieve initial fixation and allow long-term, durable fixation. The stem leverages the metaphyseal geometry of its predecessor along with GRIPTION® Porous Coating (DePuy Synthes, Warsaw, IN, USA), decreased M/L width, and a shorter distal length. A retrospective outcomes review was conducted to provide further data on the use of this femoral stem in primary cementless THA.

## Materials and methods

A retrospective outcomes review was conducted using data collected from an ongoing, standard-of-care, multi-center, company-sponsored joint replacement registry. All participating centers were in the United States. All subjects that received a TRI-LOCK BPS stem with a PINNACLE® Hip Solutions cup (DePuy Synthes, Warsaw, IN, USA) were included. Metal-on-metal articulations were excluded. Preoperative and postoperative clinical assessments included Harris Hip Score (HHS) [22], and Western Ontario and McMaster Universities Arthritis Index (WOMAC) score [23], and a registry-specific hip evaluation questionnaire.

Ethical considerations

Written informed consent was collected for all subjects prior to participation and Institutional Review Board (IRB) review and approval (approval number 20120963) were obtained for the registry and all participating sites. The central IRB from which the registry approval was obtained is WCG IRB (formerly Western Institutional Review Board {WIRB}; https://www.wcgirb.com).

Statistical analysis

Clinical assessments were summarized with sample size, mean, and standard deviation for numeric scores and with sample size and percentages for categorical responses. It is recognized that sites within the registry have different standards of care regarding clinical follow-up visits. Therefore, standardized registry visit windows were established. If an individual subject had multiple visits within the standardized window, then only the latest visit was included in the summaries.

Kaplan-Meier survivorship was performed with revision of the stem and revision of any component as endpoints. For each endpoint two survivorship analyses were performed with differing censoring assumptions. First, unrevised subjects were censored at the last clinical follow-up (clinical assumption {CA}), making no assumptions on implant survivorship beyond the patient’s last visit. Second, unrevised subjects were censored at the date of database extract (registry assumption {RA}), assuming that if a revision or death has not been reported then the devices remain implanted. In all cases survival estimates were truncated at 40 hips remaining at risk. For the survivorship of the stem component subjects were censored at the time of removal of other components.

## Results

A total of 2,040 TRI-LOCK® BPS/PINNACLE hips were implanted (excluding metal-on-metal articulation) in cementless primary THA between April 2008 and August 2017. One thousand one-hundred seventy-one (1,172; 57.5%) of the enrolled hips were female. Mean age was 65.2 years (SD 10.72, range 16 to 100) and the mean BMI was 29.2 (SD 6.00, range 14 to 68). Most primary diagnoses were osteoarthritis (1,881; 92.2%). Surgical approach was direct anterior for 1,244 (61.0%) hips; anterolateral/hardinge for 513 (25.1%) hips, posterior for 171 (8.4%) hips, and other approaches for 112 (5.5%) hips. Proximal and distal femoral bone class (as assessed and defined by the surgeon) was normal or good for 1,171 (70.9%) and 1,176 (72.2%) subjects respectively. Articulation was ceramic-on-poly in 1,295 (63.5%) cases, metal-on-poly in 681 (33.4%) and undetermined in 64 (3.1%).

There were 24 revisions (any component for any reason), 19 of which occurred prior to one year postoperatively, and 14 of 24 involving the stem. Revisions were for periprosthetic fracture (6), dislocation (5), aseptic loosening (4), infection (3), liner dissociation (1), psoas impingement (1), foreign body reaction (1), migration/loosening of the shell (1), subsidence of the stem (1), and subsidence of the stem after a fall (1). Additional details for reported revisions are shown in Table 1. Seventy-five (75) intraoperative complications were reported and included: renal events (21), cardiac events (16), hematological events (11), femoral fracture (7), respiratory events (4), neurological events (3), lack of bone stock/poor bone quality (3), femoral perforation (2), pelvic fracture (1), migration and loosening of the shell (1), foreign body reaction (1), low thyroid-stimulating hormone (TSH) levels (1), skin rash/allergic reaction (1), alcohol withdrawal (1), sickle cell issues (1), and genitourinary event (1). Fifty-six (56) study hip-related postoperative complications were reported and included: bursitis (13), infection (13; 9 superficial, 4 deep), periprosthetic fracture (6), stem loosening/migration (6), dislocation (4; 1 subject with reoccurrence), hematoma (3), heterotopic ossification (3), wound dehiscence (3), pain (2), dissociation of liner/stem subsidence (1), hip abscess (1), cellulitis (1).

**Table 1:** Revision Details

Revision Reason	Date of Surgery	Date of Revision	Time to Revision (Days)	Stem Revised	Cup Revised	Head Revised	Liner Revised
Aseptic Loosening	21-Sep-10	25-Feb-11	157	X	X	X	X
Aseptic Loosening	3-Nov-08	19-Jun-09	228	X			X
Aseptic Loosening	12-Dec-08	28-Apr-10	502	X			X
Aseptic Loosening	15-Dec-08	2-Nov-09	322	X		X	X
Dislocation	19-Dec-12	8-Mar-13	79			X	X
Dislocation	4-Oct-11	9-Dec-11	66			X	X
Dislocation	25-Oct-11	20-Dec-11	56			X	X
Dislocation	4-Jun-12	23-Oct-17	1967			X	X
Dislocation	8-May-13	19-Jun-13	42			X	X
Foreign Body Reaction	2-May-12	5-Jun-12	34			X	X
Infection	31-Jan-12	21-Feb-13	387	X	X	X	X
Infection	2-May-11	14-Nov-14	1292	X	X	X	X
Infection	10-Aug-16	27-Sep-16	48			X	X
Liner Dissociation	15-Sep-15	10-Nov-15	56			X	X
Migration/Loosening of the Shell	13-Feb-12	13-Feb-12	0		X	X	X
Periprosthetic Fracture	8-Jan-14	3-Feb-14	26	X			X
Periprosthetic Fracture	27-Sep-13	20-Feb-14	146	X		X	X
Periprosthetic Fracture	20-Nov-13	25-Nov-13	5	X			X
Periprosthetic Fracture	7-May-14	2-Jun-14	26	X			X
Periprosthetic Fracture	6-Jan-14	28-May-14	142	X			X
Periprosthetic Fracture	12-Nov-13	2-Dec-13	20	X			X
Psoas Impingement	7-Mar-13	11-Sep-13	188				X
Subsidence of Femoral Component After Fall	20-Sep-10	11-Mar-11	172	X			X
Stem Subsidence	08-Sep-14	31-Mar-21	2396	X		X	X

Survivorship

**Figure 1:**
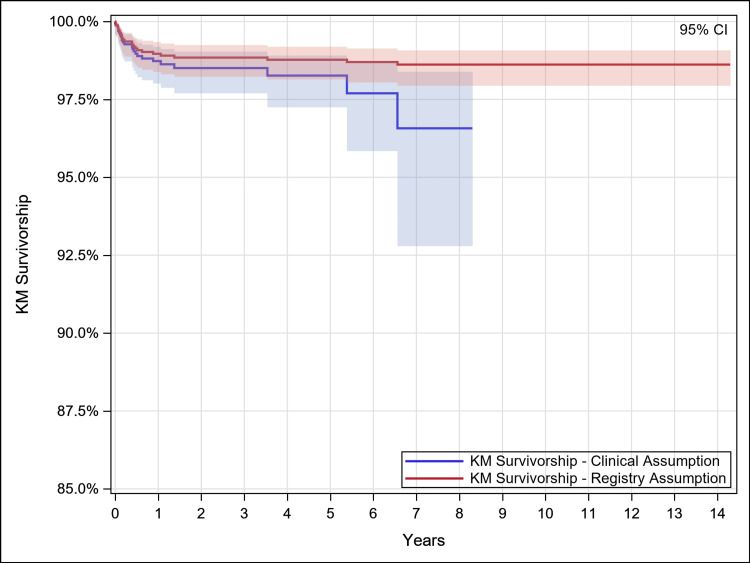
Kaplan Meier Survivorship – Any Component Any Reason

**Figure 2:**
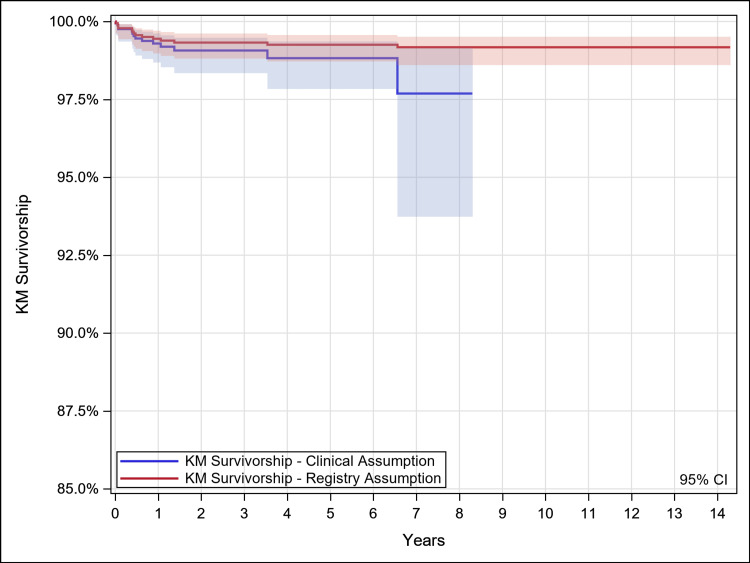
Kaplan Meier Survivorship – Stem Revision

Kaplan-Meier survivorship estimates (95% CI {confidence interval}; N with further follow-up, where N is the number of hips remaining at each post-operative interval), with survivorship defined as no revision of any component for any reason, were 96.6% (92.8%,98.4%; 45) at eight years under the clinical assumption and 98.6% (97.9%,99.1%; 90) at 14 years under the registry assumption. Additional detail is provided in Table 2 and Figure 1. With survivorship defined as stem revision for any reason, estimates were 97.7% (93.7%,99.2%; 45) at eight years under the clinical assumption and 99.2% (98.6%,99.5%; 90) under the registry assumption. Additional detail is provided in Table 3 and Figure 2.

**Table 2:** Kaplan Meier Survivorship Estimates – Any Component Any Reason

Any component any reason	0 yr	1 yr	2 yr	5 yr	7 yr	8 yr	14 yr
Hips revised	0	19	21	22	24	24	24
Hips remaining​ (CA)	2040	1086	687	231	69	45	<40
Hips remaining (RA)	2040	1710	1533	1365	1163	1046	90
CA Survival estimate ​ (95% CI)​	100.0 (100.0; 100.0)	98.7 (98.0; 99.2)	98.5 (97.7; 99.0)	98.3 (97.2; 98.9)	96.6 (92.8; 98.4)	96.6 (92.8; 98.4)	-
RA Survival estimate ​ (95% CI)​	100.0 (100.0; 100.0)	99.0 (98.4; 99.3)	98.8 (98.2; 99.2)	98.8 (98.1; 99.2)	98.6 (97.9; 99.1)	98.6 (97.9; 99.1)	98.6 (97.9; 99.1)

**Table 3:** Kaplan Meier Survivorship Estimates – Femoral Component Any Reason

Femoral component any reason	0 yr	1 yr	2 yr	5 yr	7 yr	8 yr	14 yr
Stems revised	0	10	12	13	14	14	14
Hips remaining​ (CA)	2040	1086	687	231	69	45	<40
Hips remaining (RA)	2040	1710	1533	1365	1163	1046	90
CA Survival estimate ​ (95% CI)​	100.0 (100.0; 100.0)	99.3 (98.7; 99.6)	99.1 (98.3; 99.5)	98.8 (97.8; 99.4)	97.7 (93.7; 99.2)	97.7 (93.7; 99.2)	-
RA Survival estimate ​ (95% CI)​	100.0 (100.0; 100.0)	99.4 (99.0; 99.7)	99.3 (98.8; 99.6)	99.3 (98.7; 99.6)	99.2 (98.6; 99.5)	99.2 (98.6; 99.5)	99.2 (98.6; 99.5)

Clinical outcomes

Mean total HHS (SD; N) were 52.2 (15.7; 1,836), 90.5 (11.5; 951), 91.4 (9.9; 349), 90.6 (12.2; 168) and 89.9 (18.0; 34) at pre-operative (pre-op), one year, two years, five years and 10 years post-operative (post-op) respectively. Mean WOMAC Total Scores (SD; N) were 48.8 (17.7; 1,448), 13.9 (16.1; 779), 16.4 (17.7; 303), 22.5 (21.7; 158), and 27.1 (24.2; 20) at pre-op, one year, two years, five years and 10 years post-op respectively. Additional details, including WOMAC pain, stiffness, and function scores are included in Table 4. Where thigh pain was reported by subjects post-operatively, it was present in an average of 19.8% of hips. At the first post-operative visit thigh pain was reported in 24.1% of hips, and 7.9% of hips at five years post-operatively. Additional detail regarding patient-reported thigh pain are presented in Table 5.

**Table 4:** Clinical Outcomes

	Pre-operative	< 1 year [1-303 days]	1 year [304-668 days]	2 year [669-1034 days]	3 year [1035-1399 days]	4 year [1400-1764 days]	5 year [1765-2737 days]	10 year [2738-4562 days]
Harris Hip Total Score								
Mean	52.23	87.88	90.45	91.37	89.65	90.24	90.22	90.08
SD	15.70	12.30	11.51	9.90	12.37	11.83	12.20	15.77
N	1837	1203	951	349	214	164	205	53
WOMAC Pain Score								
Mean	9.96	3.43	2.74	3.40	3.97	3.76	4.16	4.41
SD	3.81	3.57	3.70	3.95	4.29	4.27	4.53	4.55
N	1661	1096	901	349	225	178	222	49
WOMAC Stiffness Score								
Mean	4.41	2.51	1.85	2.02	2.08	2.16	2.17	2.48
SD	1.70	1.62	1.68	1.76	2.00	1.84	1.85	2.13
N	1698	1108	912	357	232	181	224	50
WOMAC Function Score								
Mean	34.42	12.52	9.57	11.54	13.09	13.76	14.45	15.63
SD	13.23	11.70	11.67	12.95	13.76	13.30	15.26	15.85
N	1480	957	794	310	207	159	205	46
WOMAC Total Score								
Mean	48.80	18.34	13.86	16.39	18.86	19.51	20.37	21.98
SD	17.66	15.96	16.07	17.66	19.43	18.52	20.88	20.63
N	1449	948	779	303	202	157	209	48

**Table 5:** Thigh Pain

	Pre-operative	< 1 year [1-303 days]	1 year [304-668 days]	2 year [669-1034 days]	3 year [1035-1399 days]	4 year [1400-1764 days]	5 year [1765-2737 days]	10 year [2738-4562 days]
None	395	597	552	222	138	96	152	43
Slight	67	88	47	16	9	5	7	4
Mild	115	46	35	12	15	5	9	1
Moderate	332	42	32	19	11	4	6	2
Marked	217	9	8	2	2	1	0	0
Totally Disabled	54	5	4	3	0	0	1	0
N Total	1180	787	678	274	175	111	175	50
Any Thigh Pain (%)	66.5%	24.1%	18.6%	19.0%	21.1%	13.5%	13.1%	14.0%
Thigh Pain >Moderate (%)	23.0%	1.8%	1.8%	1.8%	1.1%	0.9%	0.6%	0%

## Discussion

This registry evaluation of the TRI-LOCK® BPS implant shows excellent survivorship of the stem, 99.2% from 7-14 years postoperatively (RA). Stem-related complications were low, with only nine periprosthetic fractures, which all occurred within one-year post-surgery and could be due to technical complications related to stem insertion. In an observational registry data setting it is believed that RA tends to overestimate survivorship estimates, whereas CA has the potential to underestimate survivorship; we include both analysis methods in this study to improve transparency. Overall, the use of this stem was associated with good survivorship for stem and construct and a significant improvement in the HHS and WOMAC scores.

The mid-term results in our evaluation are comparable to that reported by Albers et al. who presented a 99.2% stem survivorship at five years and HHS at the final follow-up of 84.5 (SD:12.6) [24]. In a small cohort, Tsubosaka et al. reported 100% survivorship and no complications at a mean of 5.6 years [25]. The survivorship results from this evaluation are similar to those reported for the same cup and stem combination in the Australian Orthopaedic Association National Joint Replacement Registry (AOA NJRR), though our results show a slightly lower cumulative revision rate (CRR). The AOA NJRR reported a CRR at five years of 2.8% (95% CI: 1.9,4.0), and 3.3% (95% CI: 2.3,4.8) at 10 years [26]. In comparison, we saw a CRR of CRR of 1.7% (CA) and 1.2% (RA) at five years and 1.4% (RA) at 10 years.

Positive mid- to long-term survivorship has been reported in multiple systematic reviews for short metaphyseal or meta-diaphyseal fitting stems. Lidder et al. reported combined survivorship for short metaphyseal loading cementless stems of 98.6% at 12 years [27]. Tatani et al. reviewed evaluations of short femoral stems with metaphyseal or meta-diaphyseal fitting and reported survivorship of 99% at an average of 5.5 years [28]. Hauer et al. reported a median revision rate of 4.8% at 10 years for short-stem hip prostheses [29].

Incidence of thigh pain after cementless total hip arthroplasty has been reported by Brown et al. as highly variable, present in 1.9% to 40.4% of cases across a wide range of stem designs [30]. The occurrence of thigh pain for the legacy TRI-LOCK® stem was reported at 2% by Khanuja et al. [12] and 9% by Healy et al. [31]. There are also variable reports of thigh pain for the TRI-LOCK® BPS, with incidence ranging from 1.6% to 27.8% [24,32-34]. Thigh pain in our cohort was reported in 19.8% of post-operative visits. Even though most of the recorded thigh pain was “slight” or “mild” (66.6%), the overall levels of reported thigh pain from our evaluation are at the high end of the range that has been previously reported in the literature.

Limitations

Limitations of this study include a non-randomized, non-controlled design. Another limitation is the standard-of-care follow-up schedule for registry subjects, which typically does not allow the same follow-up compliance as a clinical study. Additional long-term follow-up from large national registries and clinical studies would be beneficial to continue to assess the performance of this stem.

## Conclusions

In this cohort of 2,040 wedge-shaped medial-lateral taper stems, only 14 stems were revised, which included six fractures and one stem subsidence that all occurred within the first year of implantation and could be considered a technical complication related to stem insertion. After one year, only three revisions of the stem had been reported. The overall rate of fracture was very low (0.3%). The data for this stem compares favorably to any device currently reported in the literature and can be considered a standard for comparison for proximally coated tapered-wedge stems. Additional follow-up is needed to assess long-term performance.
